# Regulatory gene network for coffee-like color morph of TYRP1 mutant of oujiang color common carp

**DOI:** 10.1186/s12864-024-10550-5

**Published:** 2024-07-02

**Authors:** Roland Nathan Mandal, Jing Ke, Nusrat Hasan Kanika, Fuyan Wang, Jun Wang, Chenghui Wang

**Affiliations:** 1 Key Laboratory of Freshwater Aquatic Genetic Resources Certificated By the Ministry of Agriculture and Rural Affairs, Shanghai Engineering Research Center of Aquaculture, National Demonstration Centre for Experimental Fisheries Science Education, Shanghai Ocean University, Shanghai, 201306 China; 2 College of Fisheries and Life Sciences, Shanghai Ocean University, 999, Huchenghuan Road, Shanghai, 201306 China

**Keywords:** TYRP1, Tyrosine metabolism, Inflammatory Autoimmune system, Cell regeneration

## Abstract

**Background:**

Neither a TYRP1-mediated highly conserved genetic network underlying skin color towards optimum defense nor the pathological tendency of its mutation is well understood. The Oujiang Color Common Carp (*Cyprinus carpio* var. *color*) as a model organism, offering valuable insights into genetics, coloration, aquaculture practices, and environmental health. Here, we performed a comparative skin transcriptome analysis on TYRP1 mutant and wild fishes by applying a conservative categorical approach considering different color phenotypes.

**Results:**

Our results reveal that an unusual color phenotype may be sensitized with TYRP1 mutation as a result of upregulating several genes related to an anti-inflammatory autoimmune system in response to the COMT-mediated catecholamine neurotransmitters in the skin. Particularly, catecholamines-derived red/brown, red with blue colored membrane attack complex, and brown/grey colored reduced eumelanin are expected to be aggregated in the regenerated cells.

**Conclusions:**

It is, thus, concluded that the regenerated cells with catecholamines, membrane attack complex, and eumelanin altogether may contribute to the formation of the unusual (coffee-like) color phenotype in TYRP1 mutant.

**Supplementary Information:**

The online version contains supplementary material available at 10.1186/s12864-024-10550-5.

## Background

Skin color is one of the highly conserved phenotypes that is not only from pigment cells or chromatophores but also affected by multiple factors, including physiological and genetic conditions [1]. In vertebrates, black and brown color, one of the dominant skin pigmentations, is mainly the consequence of a series of polymerization of derivatives of dihydroxyindole to produce insoluble melanin, which is transferred to overlying keratinocytes in the skin [2]. Changes in melanin biosynthesis can cause several physiological consequences and unusual color phenotypes. However, the genetic regulatory network underlying color phenotypes has not fully been solved [3, 4].

It is evident that tyrosinase-related protein 1 (TYRP1) has three duplicate genes resulting from whole genome duplication in the common carp genome 8.2 myr ago [5]. These three genes have multifunctional effect on melanogenesis by regulating eumelanin in quantity and aggregation [6]. It has recently been revealed that the TYRP1b gene is responsible for brown or gray skin in the color common carp variety. Multi-functionality of the TYRP1 gene on melanogenesis can influence the other pathways [6]. For example, TYRP1 has a crucial role in reducing apoptosis and oxidative stress in vivo and in vitro by elevating the expression of premelanosome protein (PMEL) [7]. On the other hand, in vertebrates, melanogenesis, taking place in mature melanocytes, is also mediated by tyrosinases (TYR), TYRP1, and dopachrome tautomerase (DCT) [8–12]. This color-generating pathway is connected with several metabolic pathways and immune systems by catabolizing two common and central metabolites, such as L-tyrosine and L-DOPA. These two metabolites can influence the interaction and coordination among different cells and components to cause skin pigmentation [3, 13].

**Fig. 1:**
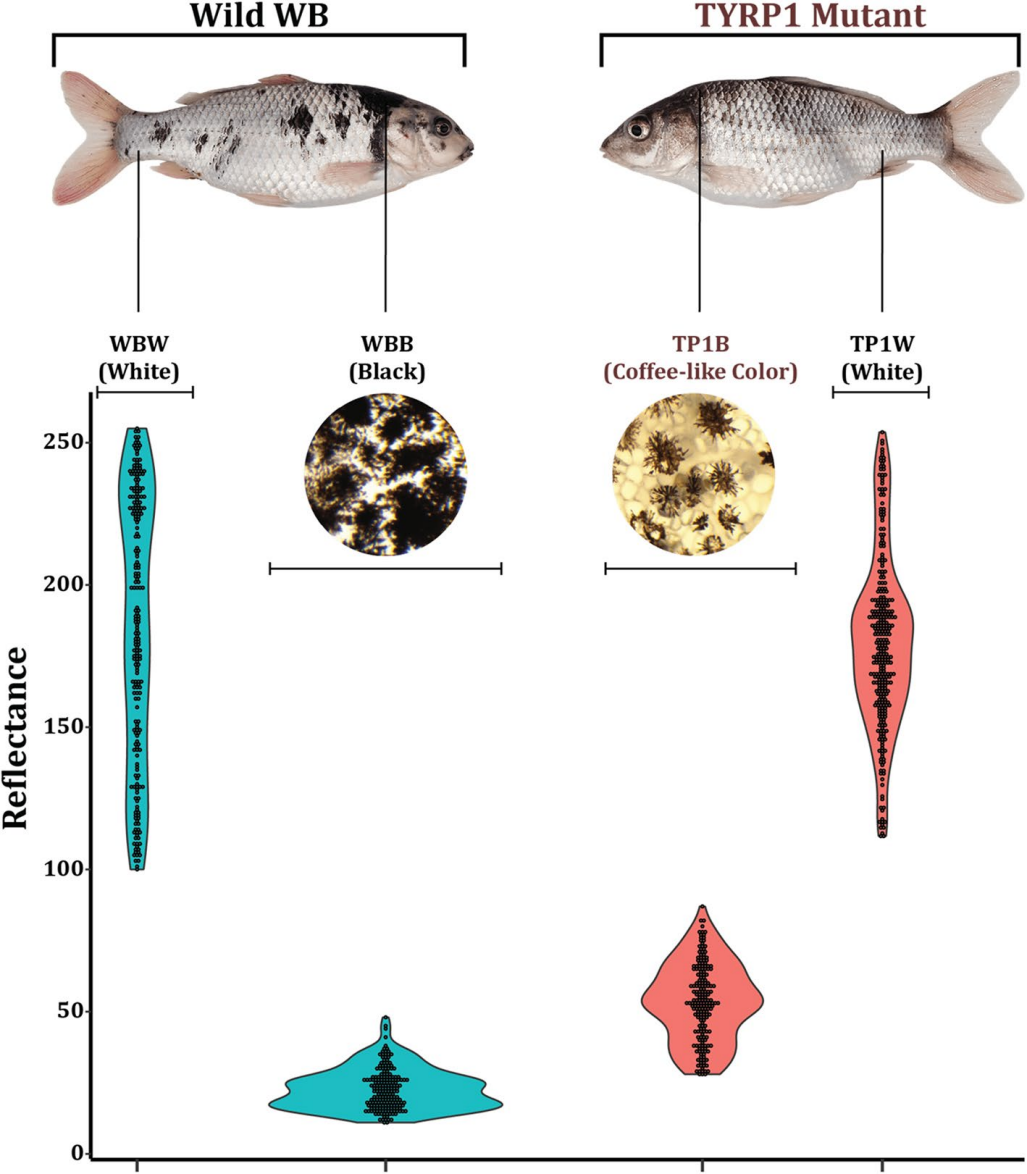
The phenotype of wild and TYRP1 mutant fishes. WBB: Black skin of wild fish; WBW: White skin of wild WB; TP1B: Coffee-like color of TYRP1 mutant; TP1W: White skin of TYRP1 mutant

The Oujiang Color Common Carp (*Cyprinus carpio* var. *color*), belonging to the Cyprinidae family, has been cultured in ponds for over 1,200 years in the Oujiang river basin of Zhejiang province in China [14]. Several variations in body color have been evident that consistently co-exist in this common carp variety. But the most predominant variety is the fishes of white skin with scattered black spots (WB), which could provide an excellent model to explore the underlying molecular mechanisms of pigment formation and development (Fig. 1). Therefore, in our previous study, TYRP1 was subjected to knocked out for revealing its function in melanin biosynthesis [6]. Our previous study found that knocking out of three TYRP1 duplicate genes produced fishes with different color phenotypes in the F0 generation [6]. In a further mating experiment involving individuals of TYRP1 F0 mutants, we observed that all the individuals were born with white skin (TP1W) with patches of unusual color phenotype (TP1B) (coffee-like) in the F1 generation. In this study, we used the next-generation sequencing technology to obtain gene expression data of the skin with coffee-like color phenotype, the white skin of TYRP1 mutant, and also the black and white skin of wild fishes of WB variety. We then used weighted gene co-expression network analysis to extract a set of highly connected genes. Both analyses enabled us to identify and characterize genes and related pathways underlying the skin of coffee-like color phenotype.

## Results

### Phenotypic characterization and the RNA-seq sequencing

We used six biological replicates for both TYRP1 mutant and wild-type fishes that were all in the same age group. The sampling conditions are given in Table S1. We found that there were four distinct color variations in TYRP1 mutant WB and wild WB. According to the color, the skin can be grouped into black skin regarded as WBB, white skin as WBW, coffee-like color as TP1B, and mutant white skin as TP1W (Fig. 1). We found that the reflection rates of WBW and TP1W are more or less similar ranging from 100 to above 250 mean RGB color channels. However, the reflection rate within 150–200 was highly frequent in case of TP1W color phenotype. On the other hand, the black skin of wild WB (WBB) exhibited reflectance of less than 50 mean RGB color channels, whereas the coffee-like skin of TYRP1 mutant WB (TP1B) showed a widely distributed reflectance ranging from 28 to 87 (Fig. 1). For this reason, we collected the transcriptome data from six biological replicates of each sample group (6 * 4 = 24 samples in total). A total of 1,327,416,078bp clean data was obtained, and the clean data of each sample reached more than 6.58Gb, and the Q30 base percentage was above 94.11% (Table S2). A total of 112,254 expressed transcripts were identified, of which 47,296 were known transcripts and 64,958 were new transcripts. A total of 58,370 expressed genes were identified in this analysis.

### Comparative differential expressed genes and pathway enrichment

**Fig. 2:**
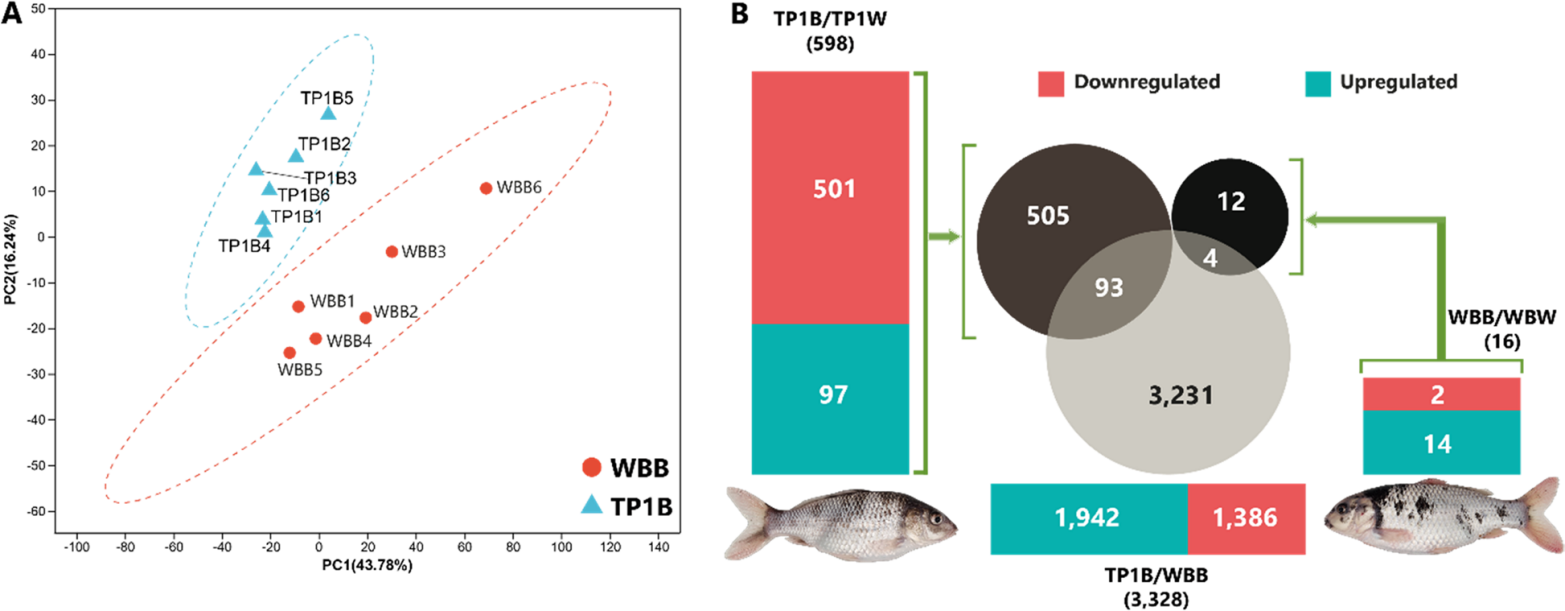
Differentiation of TP1B (Coffee-like color) and WBB (Black). **A** Differential gene expression between the TP1B and WBB; **B** Principal component analysis (PCA) of gene expression comparing TP1B and WBB

The Principal Component Analysis (PCA) shows the relationships among samples and the differences among phenotypic groups (Fig. 2A). The completely separate two clusters were evident in the comparison between TP1B (coffee-like) and WBB (black). It indicated that gene expressions were obviously different between the TYRP1 mutant and wild WB.

A total of 3,328 differentially expressed genes (DEGs) were identified in the comparison between the TP1B (coffee-like) and the WBB (black), of which 1,386 genes were upregulated, and 1,942 genes were downregulated in TP1B (Fig. 2B). Among 16 differentially expressed genes, 14 genes were upregulated, and 2 genes were downregulated in the black skin relative to the white skin of the wild fish group, whereas 97 genes were upregulated and 501 genes were downregulated in the TP1B (coffee-like) compared to the TP1W from the total 598 DEGs between two these groups (Fig. 2B).

The enrichment analysis of Gene Ontology (GO) annotation showed that in the TP1B, 42 genes were significantly upregulated relative to the TP1W in the top 30 enriched GO terms. Among these, the highest number of genes (21) encode proteins playing a crucial role in the extracellular region. And the highest number of genes (59) were significantly downregulated and are also involved in the extracellular region among the top 30 enriched GO terms (Fig. S1A, B). However, no genes in the typical melanogenesis pathway were found to be differentially expressed in TP1B relative to TP1W. On the other hand, GO enrichment profiles were completely different in the comparison between WBB and WBW, where in the WBB, several highly expressed genes were related to the pigmentation (Fig. S1C, D). Moreover, distinctly different GO terms were enriched in the comparison between the TP1B and the WBB. Most ATP-dependent activity-related genes were upregulated in the TP1B followed by peptidase inhibitor activity genes, whereas iron ion binding protein-encoding genes were significantly downregulated, followed by ATP hydrolysis activity-related genes (Fig. S1E, F).

**Fig. 3:**
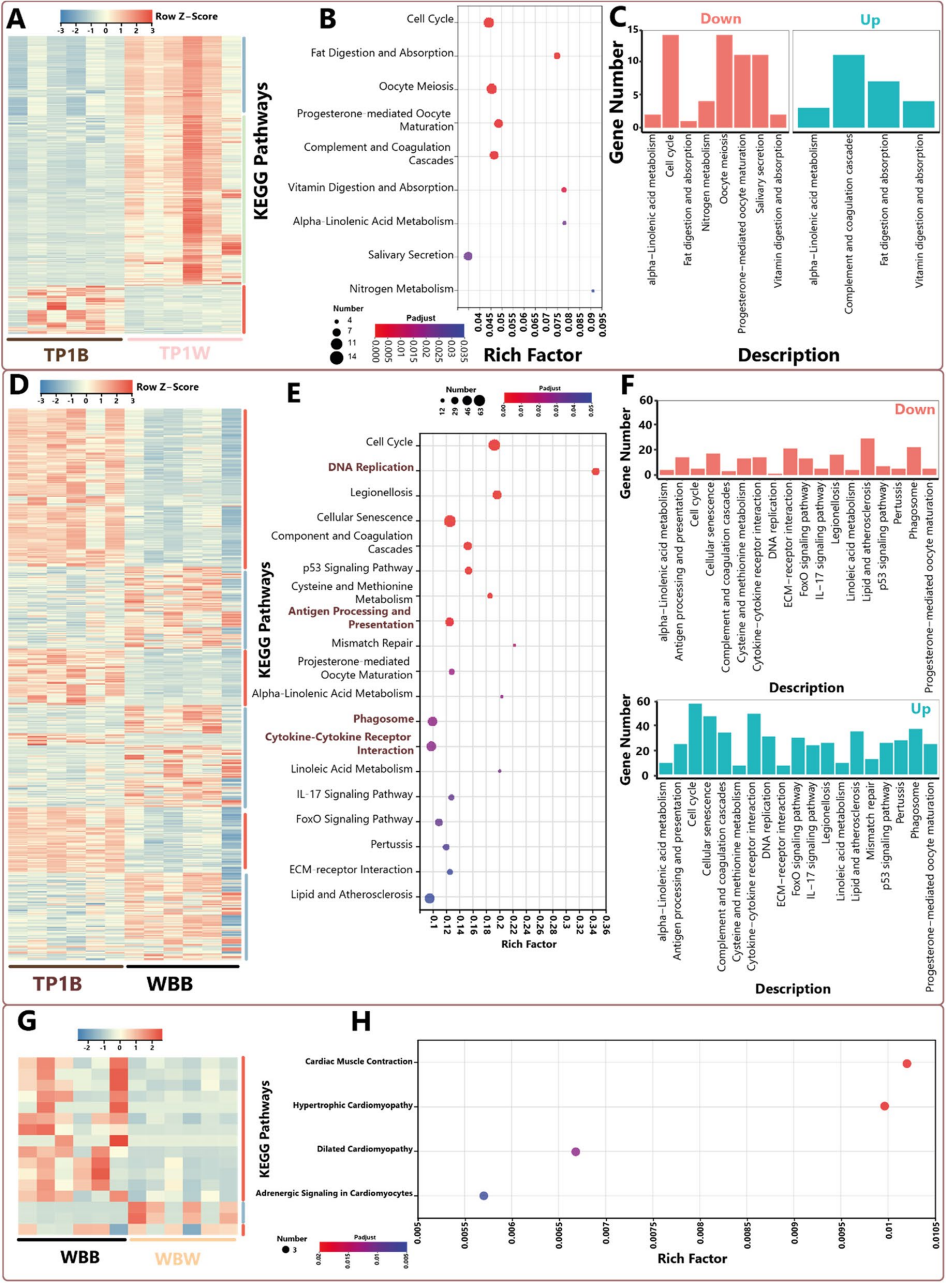
Summary of differentially expressed heatmaps and KEGG enrichment analysis. **A**-**C** Heatmap and KEGG enrichment top 20 bubble chart and the number of differentially expressed genes between the TP1B and TP1W; “Up” represents the genes that are over-expressed in the TP1B relative to TP1W; “Down” represents the downregulated genes. The vertical axis represents the KEGG Metabolic Pathways, and the horizontal axis represents the ratio of the number of genes/transcripts enriched in the KEGG Pathway to the number of annotated genes/transcripts (Background number) of the Rich factor. The larger the rich factor, the greater the degree of enrichment, the size of the dot indicates the number of genes/transcripts in this pathway, and the color of the dot corresponds to different Padjust ranges. **D**-**F** Heatmap and KEGG enrichment top 20 bubble chart against the number of differentially expressed genes between the TP1B and WBB. **G**-**H** Heatmap and KEGG enrichment top 20 bubble chart and the number of differentially expressed genes between the WBB and WBW

Regarding the KEGG pathways, in TP1B, complement and coagulation cascades-related protein-coding genes were highly expressed relative to TP1W (Fig. 3A-C). However, genes involved in the cell cycle and oocyte meiosis were noticeably downregulated. However, more pathways were enriched when we compared the gene expression profiles between TP1B and WBB (Fig. 3D-F). Among these pathways, most cell cycle-related genes were highly expressed followed by cytokine-cytokine receptor interaction genes. On the other hand, all highly expressed genes (relative to WBW) in the WBB are related to adrenergic signaling in cardiomyocytes, cardiac muscle contraction, dilated cardiomyopathy, hypertrophic cardiomyopathy, and tyrosine metabolism pathways (Fig. 3G-H).

### Significantly Co-expressed Module Genes

**Fig. 4:**
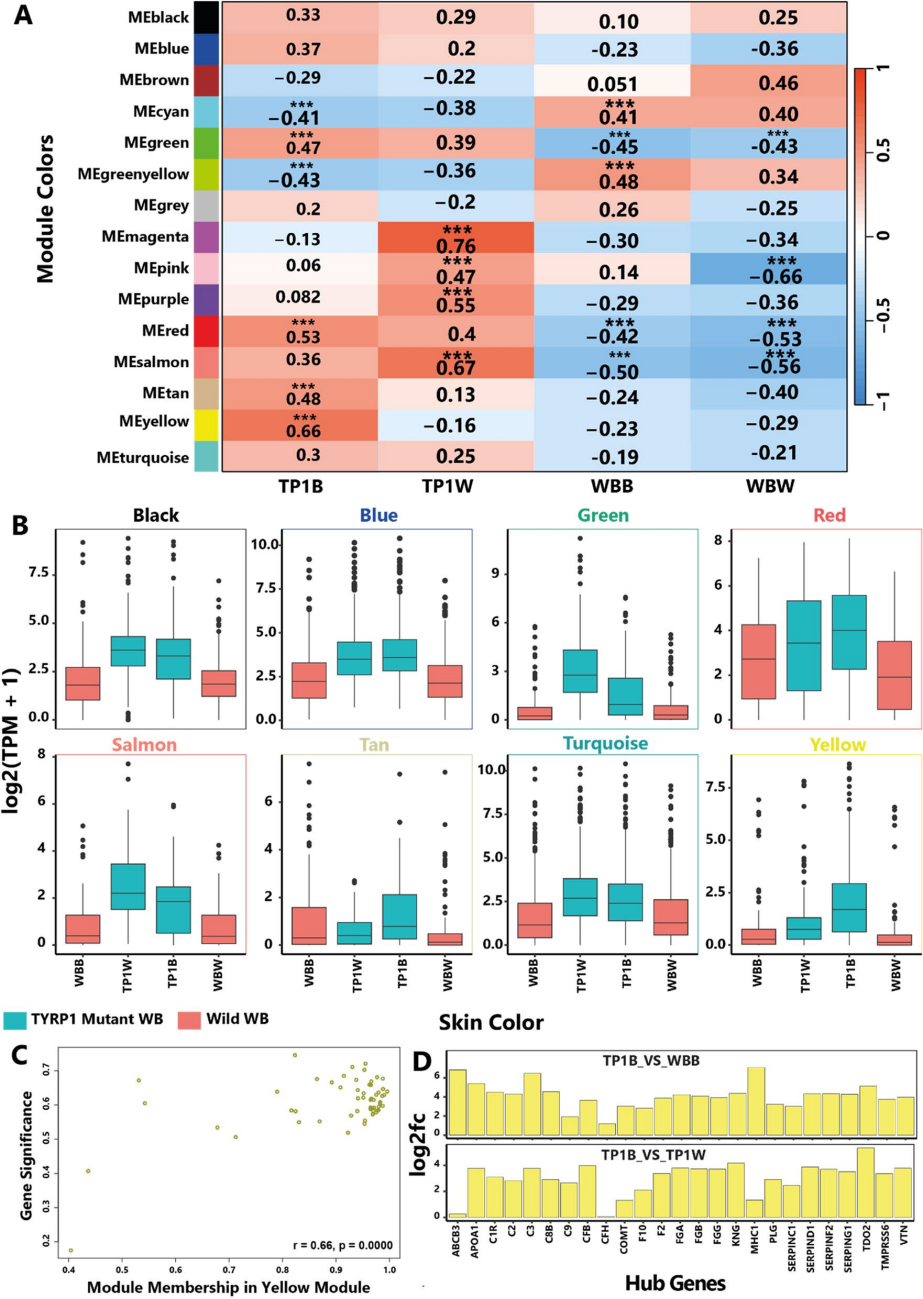
Co-expression Analysis and expression of hub genes. **A** Module-trait relationship; **B** The expression trajectory of eight modules showed a strong correlation with TYRP1 F1 mutant fishes by WGCNA analysis; **C** Gene significance to module membership in MEyellow module; **D** Log fold change of hub genes (k_ME_ ≥ 0.5) in TP1B/TP1W and TP1B/WBB comparisons

We used 710 differentially expressed genes of significantly enriched KEGG pathways (padjust ≤ 0.05) for WGCNA, and identified fifteen co-expressed modules (entitled module Eigengene (ME) (Fig. 4A; Table S3). One-way ANOVA on the mean expression of genes of identified modules showed that the expression patterns of the genes under eight modules were notable for the TYRP1 mutant fishes (Fig. 4B). Among these modules, MEyellow was the most significant module (module-trait correlation was about 0.66, *P* ≤ 0.0001) for defining TP1B (coffee-like) phenotype (Fig. 4B-C). We have shown from the differential expression analysis that the hub genes under the MEyellow module were significantly and constantly upregulated in the TP1B relative to both WBB and TP1W sample groups (Fig. 4D). According to Winden KD, we then determined the ME-based Connectivity (k_ME_) by applying the Pearson Correlation between the expression of each gene and the ME as the measure of module centrality [21].

**Fig. 5:**
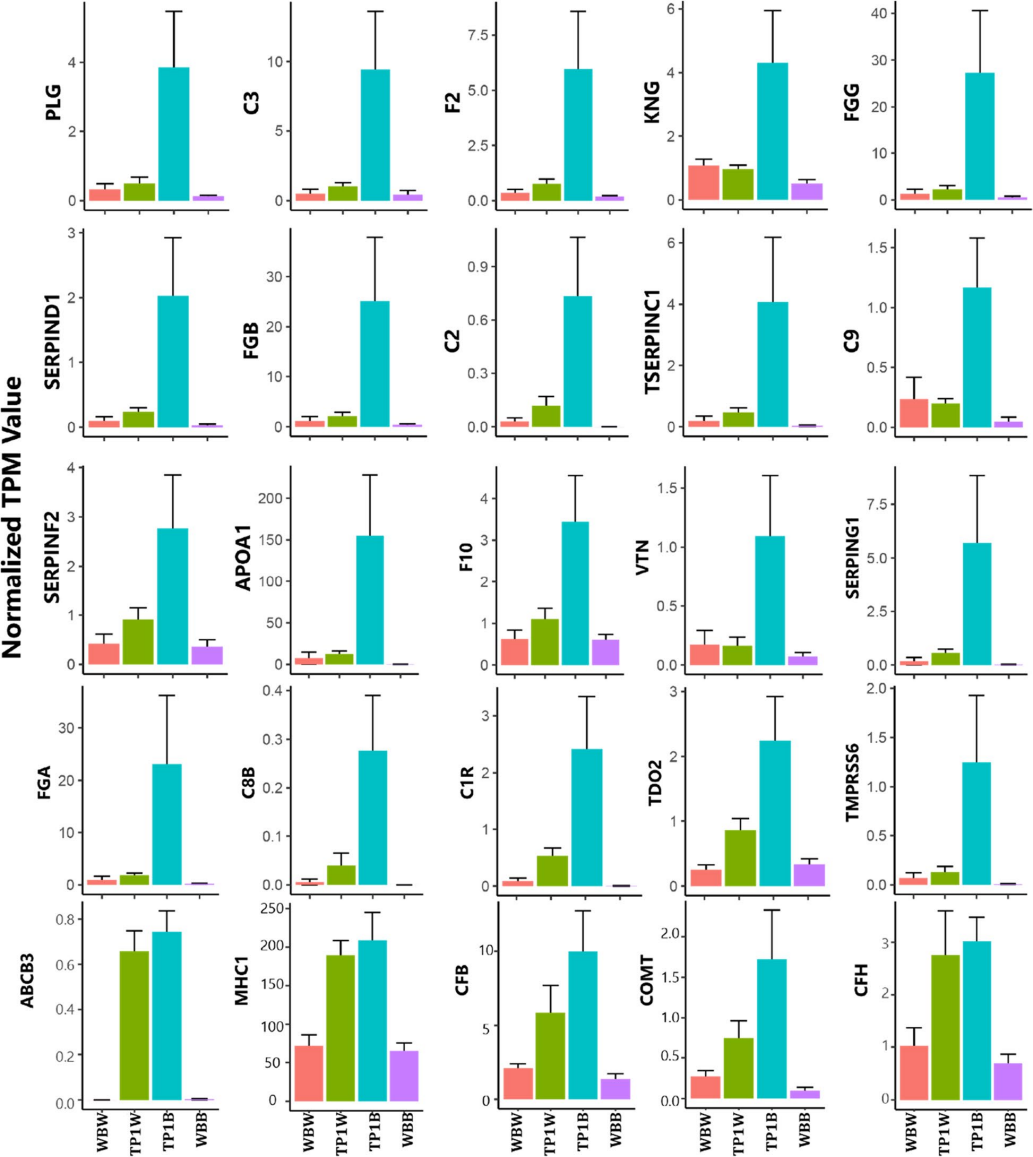
Expression variability of hub genes for the target phenotype

We found that twenty-five genes were highly correlated (≥ 0.50) with the MEyellow module, and according to k_ME_ value, thus, regarded as the hub genes [21], among which PLG was the most highly connected gene (k_ME_ = 0.999) (Fig. S2). This gene provides instructions to encode a protein called plasminogen. General linear regression analysis additionally identified statistically significant correlations (*p* < 0.0001) between MEyellow levels and the expression of hub genes across the samples (Fig. S3). We also observed noticeably higher expression of the hub genes in the TP1B compared to other phenotypes (*P* < 0.000) (Fig. 5). We also observed that the highly correlated genes to the MEblue module may have potential interactions with the hub genes of the MEyellow module to define the TP1B phenotype of the mutant fishes (see Fig. S4).

#### Pathway analysis showed upregulation of an autoimmune system

The WGCNA and KEGG pathway enrichment analysis together showed that most of the identified hub genes under the MEyellow module are related to the complement and coagulation cascades pathway (9 constantly upregulated hub genes (PLG, C3, CFB, F2, KNG, SERPINC1, SERPIND1, SERPING1, and VTN) in both TP1B-WBB and TP1B-TP1W comparisons, 8 upregulated hub genes (C2, C8B. CFH, F10, FGA, FGG, SERPINF2) in only TP1B-WBB, phagosome (4 significantly upregulated hub genes (ABCB3, C1R, C3, and MHC1) in only TP1B-WBB), and antigen processing and presentation (3 upregulated hub genes (ABCB3, MHC1, and MHC2) only in TP1B-WBB comparison, and C9 in only TP1B-TP1W). The highly correlated genes (K_ME_ ≥ 0.7) to the MEblue module are related to the cytokine-cytokine receptor interaction, and some are to the phagosome, antigen processing and presentation, and the complement and coagulation cascades pathways.

**Fig. 6:**
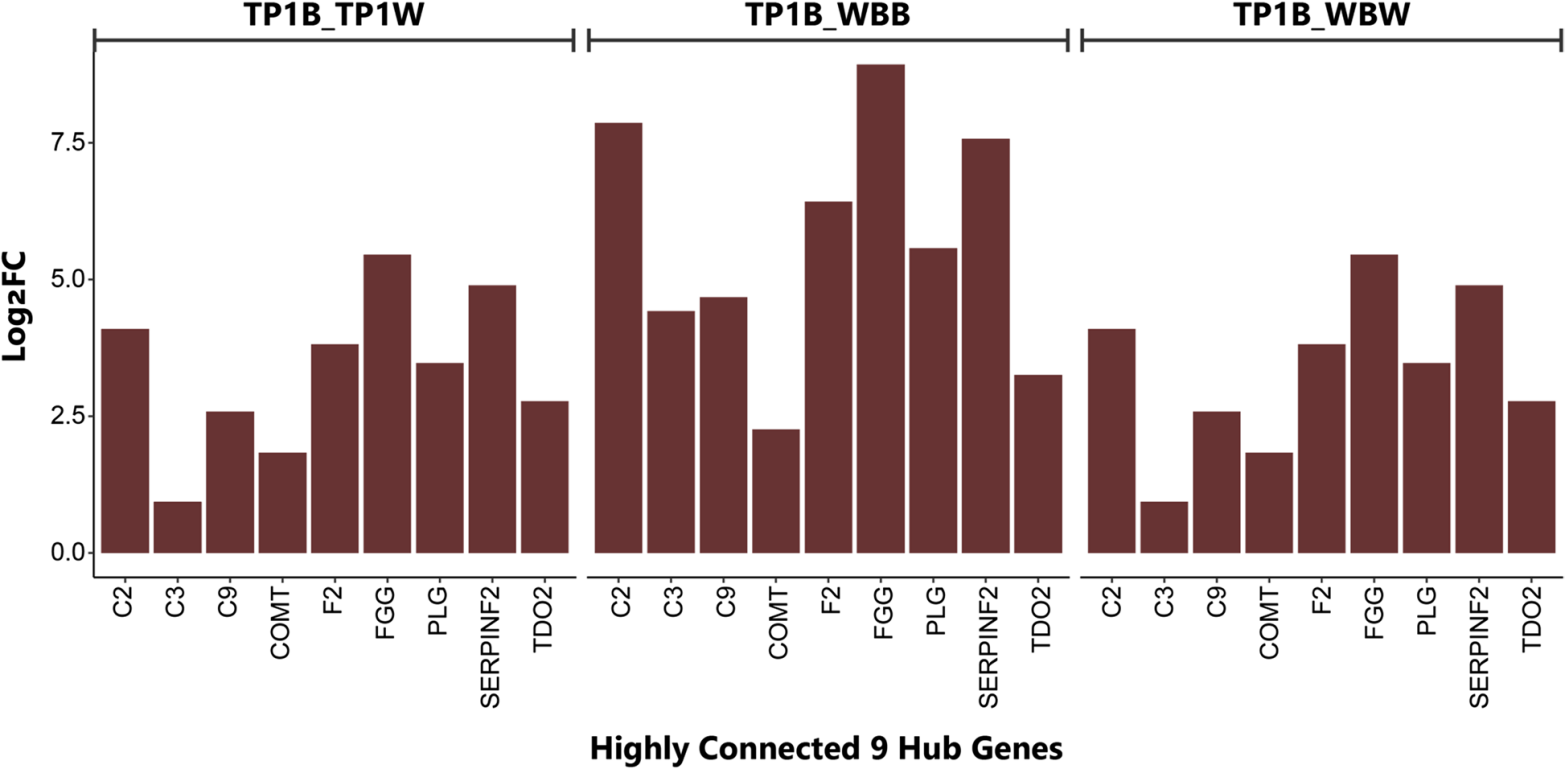
qRT-PCR validation results for the 9 highly connected hub genes

We, thus, hypothesized that the coffee-like color may indicate a heightened sensitivity to the autoimmune system in the TYRP1 mutant fishes. To validate the association of the identified hub genes with four different color phenotypes in the skin of TYRP1 mutant and wild WB fishes, we analyzed the relative expression of 9 highly connected hub genes through conducting qRT-PCR experiment. The qRT-PCR results of the 9 highly connected anti-inflammatory hub genes also show that the F2, FGG, SERPINF2 and PLG genes related to coagulation and C2 complement-related genes have been highly upregulated in the skin of TP1B relative to WBB, WBW, and even TP1B color phenotype. These results are consistent with our RNA-seq derived expressions of these genes (Fig. 6).

## Discussion

That the whole genome duplication had resulted in evolving three TYRP1 duplicate genes in the genome of common carp 8.2 myr ago was revealed in 2003 [5, 6]. Among these, mutations in the TYRP1b gene were suggested to induce grey color resulting from reducing eumelanin and brown phenotypes from eumelanin aggregation disorder [6]. Interestingly, the differentially expressed genes under the enriched GO term clearly showed a significant role of the pigmentation-related genetic mechanism underlying the reflectance rate of the WBB skin, whereas no GO term has been enriched to define the classical pigmentation process for the TP1B skin. It is, thus, expected that the coffee-like color in the TYRP1 fishes may not follow the conventional pigmentation mechanism. In the present study on the skin transcriptomes from the further mating experiment, we identified some hub genes and related pathways underlying a coffee-like color phenotype in the TYRP1 mutant fishes.

An unusual color phenotype can be formed by a number of impaired conditions caused due to melanogenesis disorder [2]. It was evident that the downregulation of TYRP1 is associated with apoptosis and oxidative stress through downregulating PMEL [7]. In the TYRP1 mutant fishes relative to wild WB fishes, the present study identified several hub genes under the MEyellow module, which are reported to eliminate apoptotic cells by constructing phagosomes [22–24]. The cells were suspected to be targeted and internalized by the upregulated toll-like receptor (TLR) and complement-1 receptor (C1R) to the immature phagosome [22–24]. The researchers think that phagosomes are matured in Endoplasmic Reticulum and Golgi complex by the upregulating histocompatibility complex class -I (MHC1) molecule, related to the cross-presentation of antigens in the TP1B [25–28]. The upregulating ATP-binding cassette transporter B family member 3 (ABCB3), having a crucial role to form a transporter in antigen presentation by MHC1 molecules, is also expected to have a role in phagosome maturation [29]. The upregulation of the myeloperoxidase encoding gene, MPO, indicated that a potent antimicrobial immune system, constituted by MPO in combination with H_2_O_2_ and chloride, had been upregulated in the TP1B [30].

COMT is demonstrated to generate metanephrine-like catecholamines for ensuring an optimum defense by regulating inflammation by preventing excessive host cell damage [31]. In the present study, overexpressed COMT indicated the probable accumulation of metanephrine in the skin of TP1B. Moreover, aggregation of upregulating COMT-mediated catecholamines can cause a long-lasting inflammatory skin disease called psoriasis, which produces red/brown-colored apoptotic cells [32]. Furthermore, upregulating COMT-mediated catecholamines has also been reported to be responsible for upregulating different inflammatory mediators [31].

**Fig. 7:**
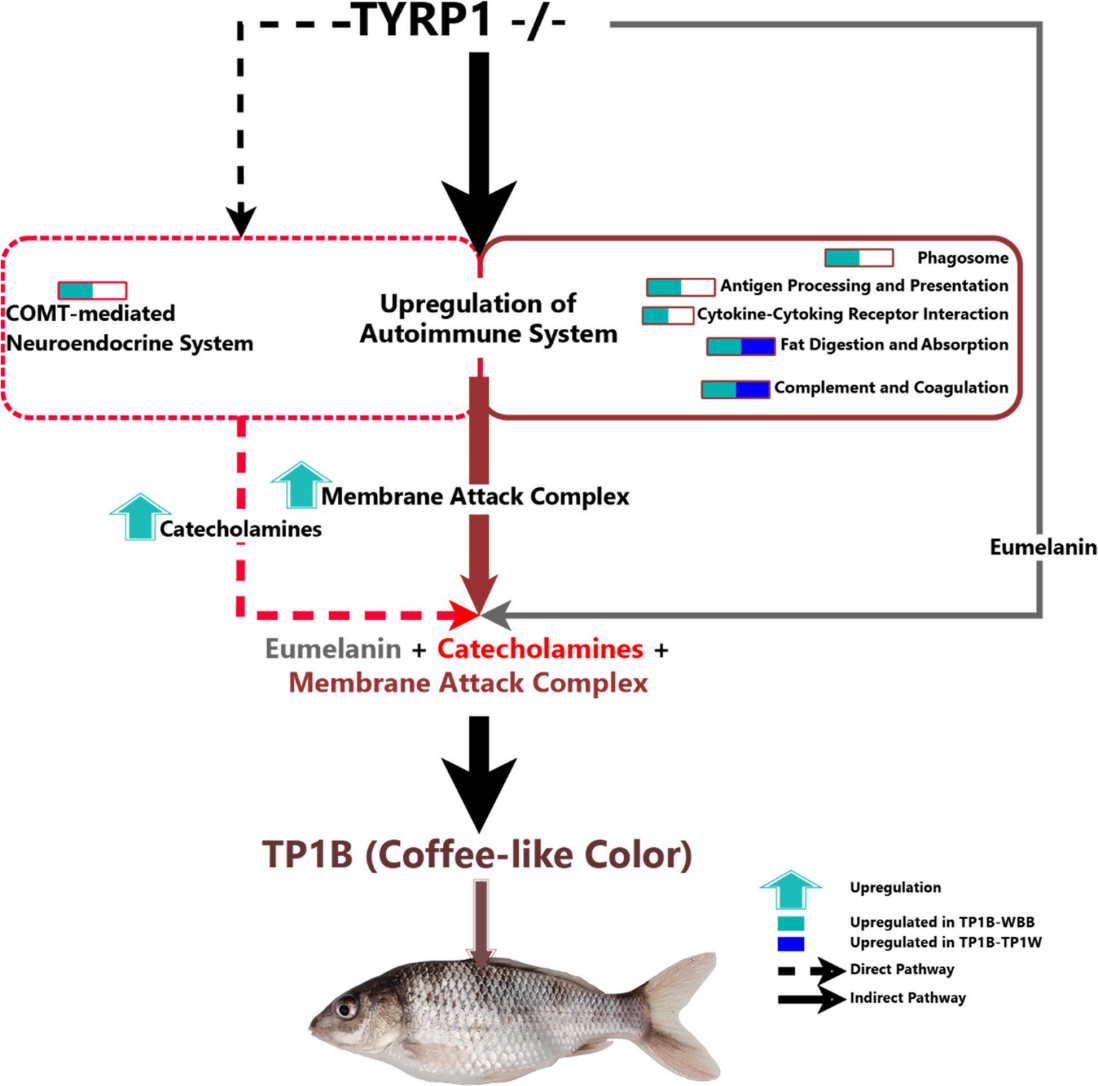
A hypothetical regulatory pathway underlying TP1B (Coffee-like) phenotype

Several studies confirmed that different inflammatory mediators have a direct and indirect relationship with skin pigmentation [33, 34]. A negative relationship has been revealed between the expression of IL‑ 17 and TNF cytokines with the levels of TYR and TYRP1 by influencing PKA and MAPK signaling pathways [35]. Moreover, some studies suggested that acute or chronic inflammation may cause psoriasis-like skin pigmentation. Similarly, we identified some highly connected genes, which are involved in a defense mechanism against inflammation called ‘Cytokine-Cytokine Receptor Interaction’. These include Interleukin 17 Receptor B (IL17RB, K_ME_ = 0.95), Tumor Necrosis Factor (TNF, K_ME_ = 0.93), Tumor Necrosis Factor Receptor-associated Factor 3 Interacting Protein 2 (TRAF3IP2, K_ME_ = 0.88) and Interleukin-1β (IL-1β, K_ME_ = 0.61). These genes are demonstrated to play a central role in innate immunity in response to pathogenic inflammatory signals and resulting stress [32, 36–39]. These inflammatory mediators can activate other leukocytes to build an adaptive and specific antibody response [31]. We also identified that the complement and coagulation pathway was highly activated by upregulating most of the 25 hub genes under the MEyellow module. It indicates that the skin with the coffee-like color had required a set of genes to be crucially involved in the inflammatory response. This pathway may build a defense line of innate immunity by complement and hemostasis by coagulation system [40]. There are three reported traditional pathways of complement activation featuring classical pathway initiated by antibody-antigen complex, a lectin-dependent pathway by mannan-binding lectin-carbohydrate complexes, and an alternative pathway by microbial surfaces [41]. In the present study, we identified that all the complement pathways had become active to opsonize and remove the apoptotic cells. In addition to making an innate immunity, complement is now thought to be involved in tissue regeneration [42] and clearance of debris [43]. In the present study, because of being highly connected hub genes complement C3 is expected to play a central role. According to the observation of Clark A., et al. [42], it is suspected that complement C3 became active via plasmin (PLG) and thrombin (F7, F10, F2) proteases in a regenerative response. Regenerative effects of this pathway have also been evident in neurons [44], osteoblasts [45], and dental pulp progenitors [46]. It is, thus, expected that upregulation of C3 from the alternative pathway, C2 from both classical and lectin-dependent complement pathways highly increased the proteolytic cleavage of C3 to form C3b for mediating target clearance and forming C5 to C9 by associating with C3 convertase and finally initiating the assembly of the membrane attack complex as the regenerated cells. These complement proteins are reported to generate a complex color for the membrane attack complex. For example, C8B, a highly expressed hub gene, looks red-color, whereas C9 looks light blue color [47]. Since the C9 protein was not highly expressed in the present study, we expected that in the membrane attack complex, the red color became highly dominant which was consistent with the mean of mean reflectance ratio of the TP1B skin color in the R:G:B color channels (2:1:1). It is, thus, expected that aggregation of catecholamines, membrane attack complex, and reduced amount of eumelanin results in generating red, red with blue and brown/grey color respectively and, thus, deriving the coffee-like color in the TYRP1 mutant fishes (Fig. 7).

One of the limitations of our study is that we did not consider the metabolites that may act as confounding factors for the expression of the regulatory gene network [48]. An integrated metabolic pathway of differentially expressed hub genes and differential metabolites can explain how the hub genes regulate the coffee-like color phenotype. Moreover, the fishes selected for the gene expression study were classified only by microscopic observation, but immunohistological investigations were not conducted to identify COMT-mediated neurotransmitters and inflammatory proteins in the sample group of fish. It is, thus, possible that the skin without coffee-like color (TP1W) may have had neurotransmitters and inflammatory molecules that could not be determined by microscopic observation. Therefore, further studies with detailed immunohistological investigation will be required to associate these molecular and physiological changes.

## Conclusions

Our findings provide an idea for the genetic network underlying the coffee-like color morph of the TYRP1 mutant of Oujiang Color Common Carp. In our hypothesis, the coffee-like color in the skin of TYRP1 mutant fish was generated by upregulating a regulatory gene network of an autoimmune system in response to inflammation. It is concluded that a network of genes involved in the biosynthesis and metabolism of catecholamine, membrane attack complex, and eumelanin may derive the coffee-like color in the TYRP1 mutant fishes.

## Methodology

### Fish Sampling and Ethical Statement

In this study, the fish were reared in a rearing pond with controlled water conditions and quality at the Aquatic Animal Genetic Resource Station of Shanghai Ocean University (Shanghai, China). Both TYRP1 mutant (TYRP1^−/−^), and wild type of white with black variety (WB, TYRP1^+/+^) fishes were selected for the study. We followed a conservative approach to characterize the fish groups into four color phenotypes: TP1B—skin of coffee-like color of TYRP1 mutant, TP1W—white skin of TYRP1 mutant, WBB—black skin of wild WB, and WBW—white skin of wild WB (Fig. 1). The TYRP1 gene was knocked out using CRISPR Cas9 technology, which resulted in producing fishes with different color phenotypes, including fishes without black patches from the wild WB. These mutant fishes had been reared separately in a rearing pond for two years to make them subjected to further mating experiments. Skin transcriptome data were collected from the skin of four types of mentioned color phenotypes separately from six biological replicates. The sample fishes were temporarily reared and starved in containers for twenty-four hours before dissection. Before dissecting, anesthesia was administered with methane sulfonate (MS222). The dissected skins (2-3cm^2^ in size) were then rapidly poured into liquid nitrogen and stored in a refrigerator at -80℃ temperature for RNA extraction. The research was performed in agreement with the guidelines on the ethical use of animals for scientific purposes set forth by the Institutional Animal Care and Use Committee (IACUS) of Shanghai Ocean University. To ensure proper care and use of animals, the sampling procedure abides by the IACUS guidelines and ICLAS ethical guidelines. We measured the reflectance of the four skin samples separately based on the photographs, according to Schneider, C.A. et al. [15].

### RNA isolation, library preparation, and transcriptome sequencing

The total of 24 biological replicates were selected for RNA-seq. TRIzol® reagent was used to extract total RNA. The quality of the RNA was assessed using an Agilent 2100 Bioanalyzer. We monitored the RNA degradation and contamination via 1% agarose gel. To construct the sequencing library High-quality RNA samples (OD260/280 = 1.8 ~ 2.2, RIN ≥ 8.0, > 1μg) were selected. RNA-seq libraries were quantified using TBS380 and sequenced on the Illumina Hiseq 4000 platform (paired-end mode, 2 × 150 bp). To ensure high-quality sequencing data, the raw reads were filtered using SeqPrep software (https://github.com/jstjohn/SeqPrep) and Sickle software (https://github.com/najoshi/sickle). The filtering process involved the removal of adapter sequences, trimming of reads at the 3' end with a quality score below 30, removal of reads with more than 10% Ns, and exclusion of reads shorter than 50 bp. The clean reads of each sample were mapped to the assembled *Cyprinus carpio* genome (https://www.ncbi.nlm.nih.gov/genome/?term=common+carp) using Hisat2 2.2.1 [16] and the alignment rate ranged from 77.38% to 88.08%. Based on the existing reference genome mapped reads were assembled and spliced by using the software StringTie (http: ccb.jhu.edu/software/stringtie/) [17]. For gene functional annotation, genes were annotated by using BlastX searches against three protein databases, including the NCBI non-redundant (nr) database, the UniProt database, and the Ensembl zebrafish protein database, with an E-value cutoff of 1e^−10^.

### Differential expression and enrichment analysis

The histat2-stringtie pipeline was followed to measure differential expressed genes (DEGs) [18]. Clean reads at first were mapped to our assembled *Cyprinus carpio* var. color genome by using Hisat2-2.1 software. The Stringtie v1.3.4 software was then used to calculate the expected TPM for the skin of each color phenotype (WBB, WBW, TP1B, and TP1W). Finally, the DESeq2 R-software package was applied to identify differentially expressed genes (DEGs) with fold change > 2 and p ≤ 0.05. The clusterProfiler 4.6.0 software was used to conduct GO enrichment analysis [19].

### Gene co-expression analysis

An unbiased WGCNA co-expression network analysis was performed on the DEGs, which are involved in the significantly enriched KEGG pathways. To avoid any over-simplification of the unusual color phenotype (coffee-like color), we applied Weighted Gene Co-expression Network Analysis (WGCNA) to identify a set of co-expressed genes (here called ‘Module’ in the analysis) underlying the major functional elements. These elements were supposed to play an important role in changing gene expression in the TP1B (coffee-like) phenotype and were considered hub genes that are central to the biological pathways. Based on a fit-to-scale-free topology, we computed a weighted signed network with a threshold soft power of 7. To define modules, a topological overlap dendrogram was used with a minimum module size of 30 genes. Module and trait associations were determined by calculating the correlation coefficient (K_ME_) between module eigengene (ME) and traits. Cytoscape v3.10 software was used to analyze and visualize the Gene co-expression networks for the selected modules [20]. We used the STRING database to identify the protein–protein interaction network (binding affinity and co-expression scores) between important inter-related pathways.

### Supplementary Information

Supplementary Material 1.

## Data Availability

The raw sequencing reads for the skin transcriptome are available in CNCB (China National Center for Bioinformation) sequence read archives under the Project accession number PRJCA018085 (https://bigd.big.ac.cn/gsa/browse/CRA011875).
