# Supplementary material for: Regulatory gene network for coffee-like color morph of TYRP1 mutant of oujiang color common carp
Source: BMC Genomics. 2024 Jul 2;25:659. doi: 10.1186/s12864-024-10550-5 (PMC11218255; doi:10.1186/s12864-024-10550-5)
Supplement: Supplementary file 1 — Supplementary Material 1. [file 12864_2024_10550_MOESM1_ESM.docx]

# Supplementary Figures


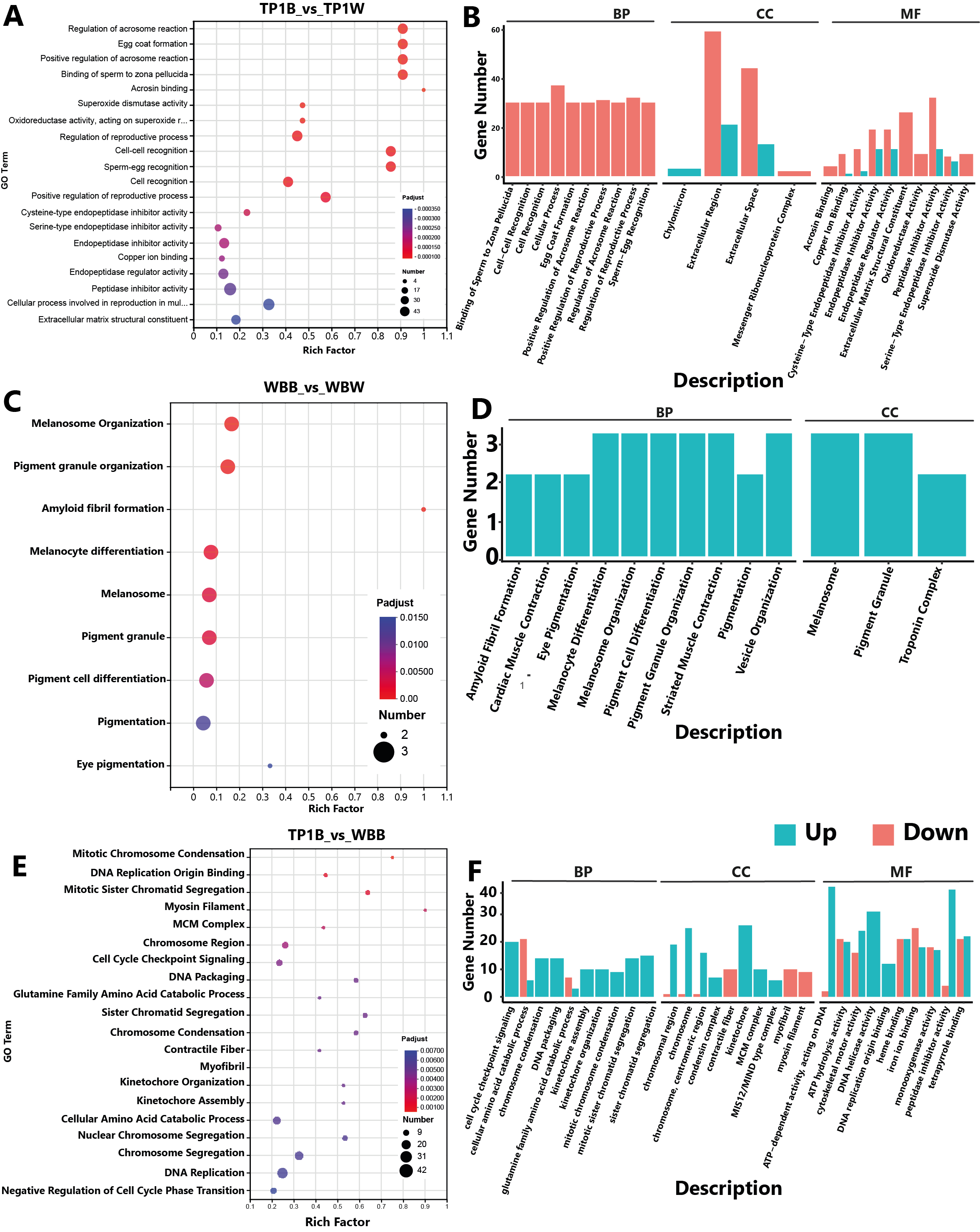


**Fig. S1** Summary of GO enrichment analysis. **A-B** GO enrichment top 20 bubble chart and Gene Ontology Classification of differentially expressed genes between the TP1B and TP1W; “Up” represents the genes that are over-expressed in the TP1B relative to TP1W; “Down” represents the downregulated genes. The vertical axis represents the GO Term, and the horizontal axis represents the ratio of the number of genes/transcripts enriched in the GO term to the number of annotated genes/transcripts (Background number) of the Rich factor. The larger the rich factor, the greater the degree of enrichment], the size of the dot indicates the number of genes/transcripts in this GO Term, and the color of the dot corresponds to different Padjust ranges. **C-D** GO enrichment top 20 bubble chart and Gene Ontology Classification of differentially expressed genes between the WBB and WBW. **E-F** GO enrichment top 20 bubble chart and Gene Ontology Classification of differentially expressed genes between the TP1B and WBB.


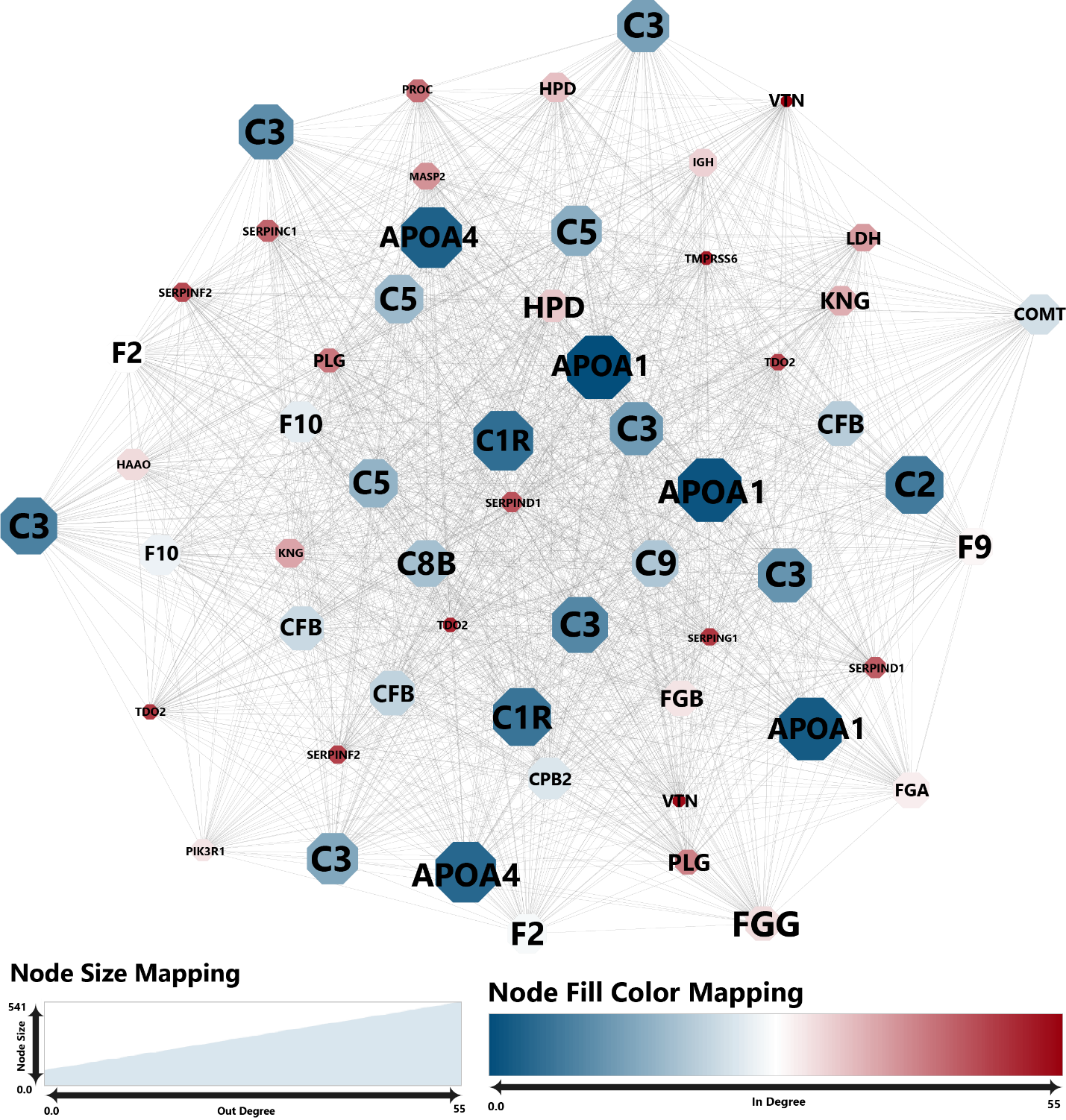


**Fig. S2** Regulatory protein network for MEyellow module


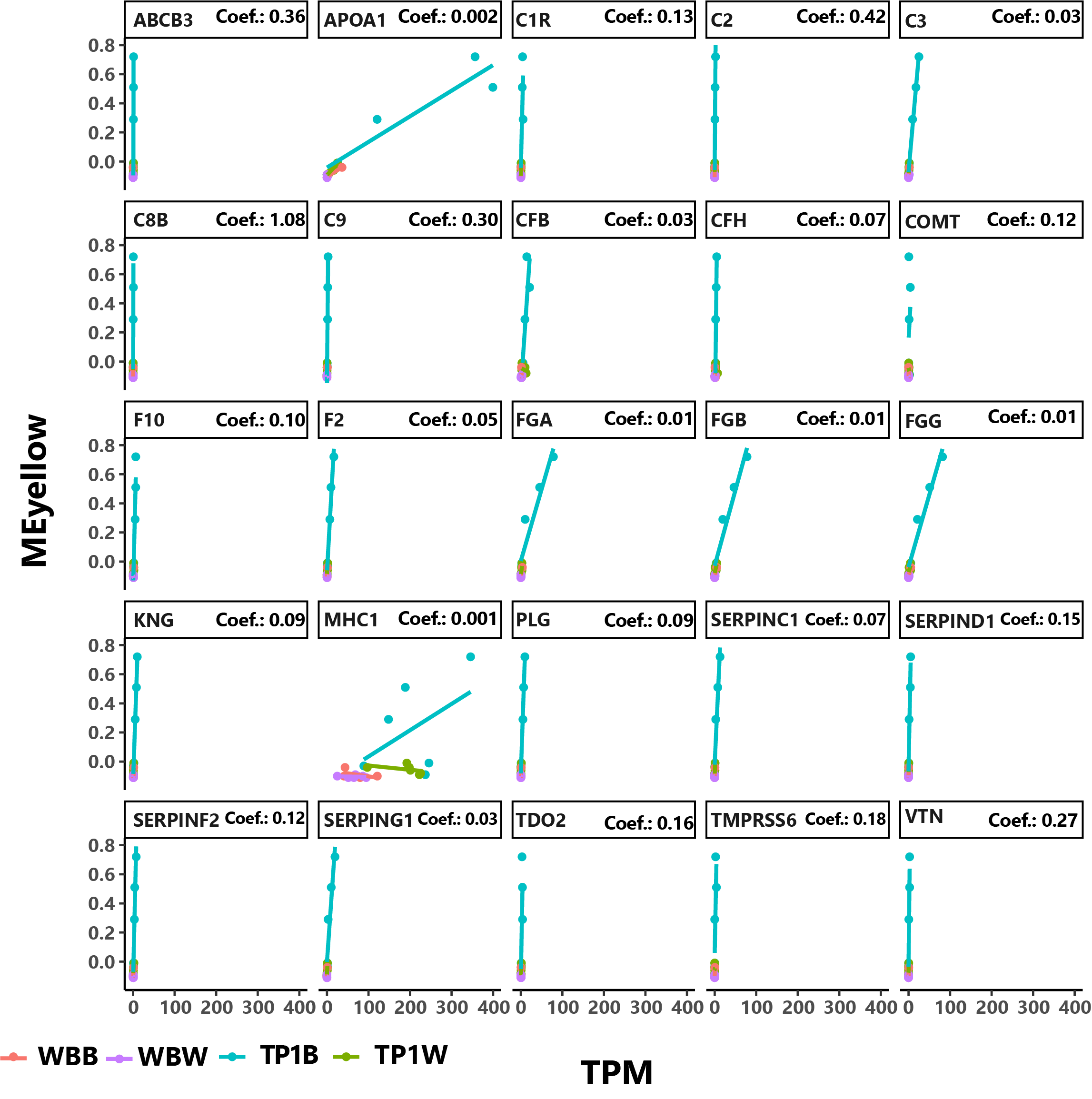


**Fig. S3** Expression of hub genes towards network centrality


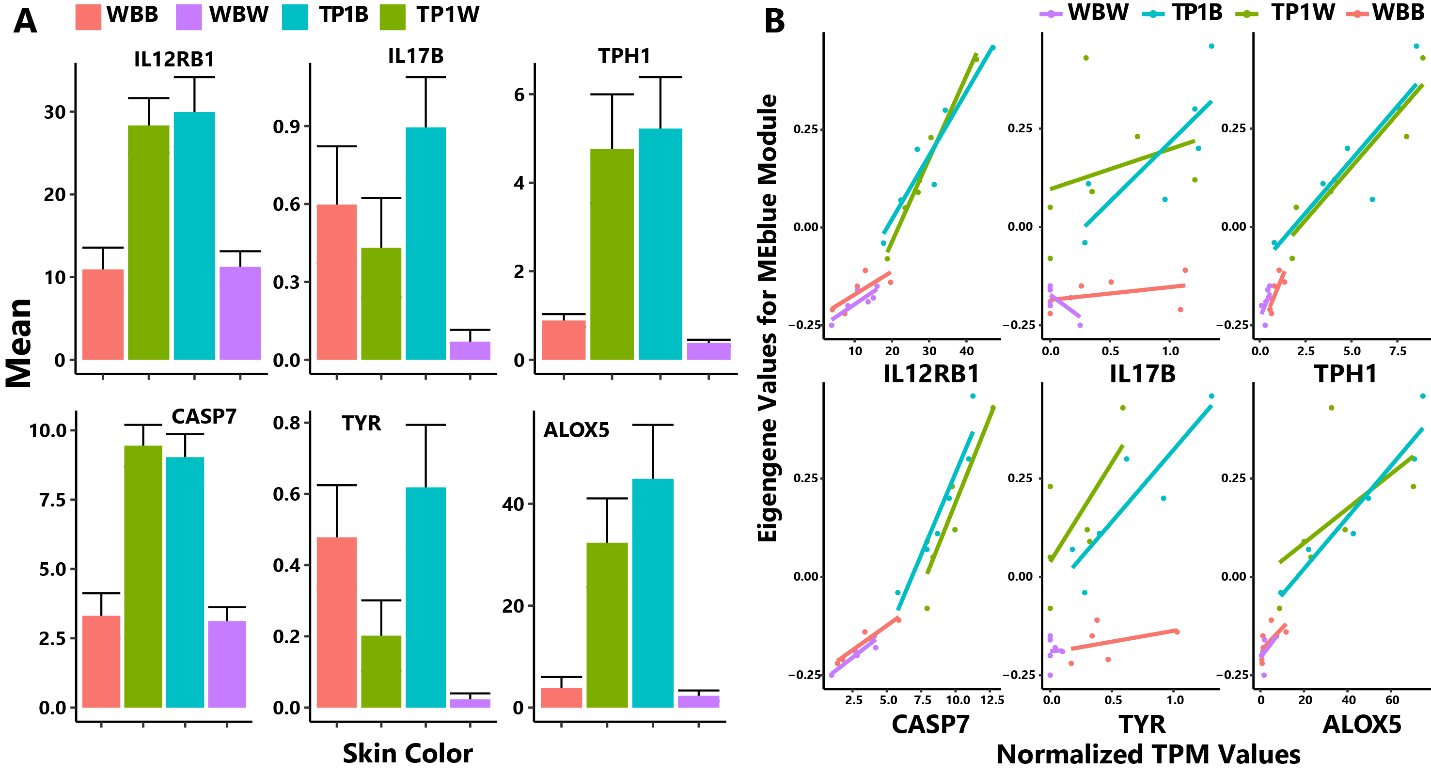


**Fig S4** Hub genes for MEblue module. **A** Expression of major six hub genes (K_ME_ ≥ 0.95); **B** Linear regression between the expression and the Eigengene value

# Supplementary Tables

Table S1: Sampling conditions of biological replicates of TYRP1 mutant and wild-type fishes

| **Strain** | **Total Length (cm)** | **Standard Length (cm)** | **Body Weight (gm)** | **Age (Year)** | **Replicate Number** |
| --- | --- | --- | --- | --- | --- |
| **Wild WB** | 24.73±3.51 | 19.43±2.78 | 192.23±60.57 | 1 | 6 |
| **TYRP1 Mutant** | 16.97±1.17 | 13.80±0.91 | 130.34±9.00 | 1 | 6 |

Table S2: Raw sequencing data of TYRP1-mutant and Wild Fish Group

| **Fish Strain** | **Replicates** | **Raw Reads** | **Clean Reads** | **Mapped Reads** | **Mapping Ratio (%)** | **GC Content (%)** |
| --- | --- | --- | --- | --- | --- | --- |
| TYRP1 Mutant | Coffee-like Color Morph | 345,143,038 | 343,205,580 | 288,572,649 | 84 | 47.76 |
|  | White Skin | 332,220,400 | 330,486,424 | 283,091,348 | 86 | 47.78 |
| **Sub-total =** | | **677,363,438** | **673,692,004** | **571,663,997** | **85** | **47.77** |
| Wild Fish | Black Skin | 350,346,342 | 348,368,744 | 299,802,296 | 86 | 48.28 |
|  | White Skin | 307,166,946 | 305,355,330 | 265,763,957 | 87 | 47.99 |
| **Sub-total =** | | **657,513,288** | **653,724,074** | **565,566,253** | **87** | **48.13** |
| **Grand Total =** | | **1,334,876,726** | **1,327,416,078** | **1,137,230,250** | **86** | **47.95** |

**Table S3: Eigengene values of different modules for different samples**

| **Fish Strain** | **Skin Colour** | **Sample** | **Pink** | **Purple** | **Magenta** | **Cyan** | **Brown** | **Greenyellow** | **Tan** | **Green** | **Black** | **Salmon** | **Blue** | **Turquoise** | **Red** | **Yellow** | **Grey** |
| --- | --- | --- | --- | --- | --- | --- | --- | --- | --- | --- | --- | --- | --- | --- | --- | --- | --- |
| **TP1** | **Unusual Colour**  **Coffee-like color**  **TP1B** | **1** | -0.12 | -0.18 | -0.10 | -0.08 | -0.15 | -0.17 | -0.06 | -0.07 | -0.07 | 0.02 | 0.30 | 0.12 | -0.05 | -0.09 | -0.13 |
|  |  | **2** | -0.13 | -0.06 | -0.11 | -0.20 | -0.17 | -0.15 | -0.02 | -0.04 | -0.13 | 0.02 | 0.07 | 0.08 | 0.02 | 0.51 | -0.28 |
|  |  | **3** | -0.13 | -0.14 | -0.12 | -0.09 | -0.22 | -0.13 | 0.45 | -0.06 | -0.09 | 0.01 | 0.46 | 0.09 | 0.21 | -0.01 | -0.10 |
|  |  | **4** | -0.13 | -0.20 | -0.15 | -0.11 | -0.19 | -0.13 | -0.02 | -0.04 | 0.18 | 0.10 | 0.11 | 0.29 | -0.05 | 0.72 | 0.09 |
|  |  | **5** | -0.07 | -0.01 | -0.03 | -0.26 | -0.11 | -0.17 | 0.12 | -0.04 | -0.04 | -0.07 | -0.04 | -0.05 | 0.75 | 0.29 | -0.12 |
|  |  | **6** | -0.13 | -0.18 | -0.09 | -0.12 | -0.14 | -0.17 | -0.04 | -0.03 | 0.28 | 0.09 | 0.20 | 0.23 | 0.14 | -0.03 | -0.10 |
|  | **Colourless**  **TP1W** | **1** | -0.13 | -0.18 | -0.12 | -0.08 | -0.16 | -0.10 | -0.09 | 0.22 | 0.22 | 0.08 | 0.23 | 0.19 | -0.10 | -0.09 | -0.23 |
|  |  | **2** | -0.06 | -0.02 | -0.01 | -0.15 | -0.05 | -0.16 | -0.09 | 0.04 | -0.08 | 0.00 | 0.05 | 0.07 | 0.41 | -0.04 | -0.04 |
|  |  | **3** | -0.13 | -0.13 | -0.08 | -0.06 | -0.16 | -0.06 | -0.05 | 0.22 | 0.19 | 0.10 | 0.43 | 0.22 | -0.05 | -0.08 | -0.03 |
|  |  | **4** | -0.09 | 0.08 | -0.11 | -0.18 | -0.16 | -0.16 | -0.07 | 0.82 | 0.23 | 0.05 | -0.08 | 0.34 | 0.02 | -0.06 | 0.33 |
|  |  | **5** | -0.09 | -0.04 | -0.10 | -0.14 | -0.20 | -0.13 | -0.08 | 0.23 | 0.10 | 0.03 | 0.09 | 0.20 | 0.03 | -0.01 | 0.11 |
|  |  | **6** | -0.12 | -0.12 | -0.04 | -0.18 | -0.09 | -0.15 | -0.05 | 0.08 | 0.33 | 0.89 | 0.12 | 0.37 | -0.03 | -0.04 | -0.12 |
| **WB** | **Black**  **WBB** | **1** | 0.01 | 0.07 | 0.31 | 0.32 | 0.14 | 0.08 | -0.08 | -0.11 | 0.30 | -0.11 | -0.11 | -0.15 | -0.07 | -0.11 | 0.44 |
|  |  | **2** | 0.01 | 0.02 | -0.11 | 0.01 | -0.04 | -0.03 | 0.82 | -0.11 | 0.22 | -0.12 | -0.21 | -0.22 | 0.01 | -0.10 | 0.12 |
|  |  | **3** | 0.26 | 0.59 | -0.05 | -0.05 | 0.01 | 0.04 | -0.07 | -0.12 | 0.19 | -0.12 | -0.22 | -0.22 | -0.09 | -0.04 | -0.09 |
|  |  | **4** | 0.00 | -0.07 | 0.01 | 0.45 | 0.19 | 0.24 | -0.08 | -0.13 | -0.20 | -0.11 | -0.15 | -0.21 | -0.17 | -0.10 | -0.25 |
|  |  | **5** | -0.07 | -0.16 | -0.06 | 0.08 | 0.16 | 0.62 | -0.08 | -0.12 | -0.24 | -0.11 | -0.14 | -0.17 | -0.16 | -0.10 | -0.04 |
|  | **White**  **WBW** | **1** | -0.05 | -0.12 | 0.83 | 0.15 | 0.44 | 0.09 | -0.06 | -0.12 | -0.22 | -0.12 | -0.16 | -0.17 | -0.18 | -0.09 | 0.34 |
|  |  | **2** | 0.13 | 0.24 | 0.00 | 0.13 | 0.06 | -0.02 | -0.09 | -0.12 | -0.22 | -0.14 | -0.20 | -0.21 | -0.15 | -0.11 | -0.03 |
|  |  | **3** | 0.84 | 0.08 | 0.24 | -0.01 | 0.39 | 0.17 | -0.08 | -0.13 | -0.27 | -0.13 | -0.19 | -0.20 | -0.16 | -0.10 | 0.02 |
|  |  | **4** | -0.06 | -0.20 | -0.17 | -0.08 | -0.20 | -0.12 | -0.09 | -0.14 | -0.22 | -0.14 | -0.25 | -0.24 | -0.19 | -0.10 | -0.36 |
|  |  | **5** | 0.21 | 0.32 | 0.02 | 0.05 | 0.37 | 0.49 | -0.10 | -0.12 | -0.26 | -0.11 | -0.18 | -0.20 | -0.10 | -0.11 | 0.10 |
|  |  | **6** | 0.05 | 0.43 | 0.07 | 0.61 | 0.31 | 0.11 | -0.10 | -0.10 | -0.20 | -0.12 | -0.15 | -0.16 | -0.07 | -0.11 | 0.37 |

Table S4: Primers for RT-qPCR validation investigation of 9 anti-inflammatory hub genes

| **Gene Name** | **Primers** | **Strand** |
| --- | --- | --- |
| C2 | CCAGTTCGTCAAATCGTGCT | Forward |
|  | TGTGCTCAGGAATGGTGGAT | Reverse |
| C3 | GAGGGCAGAATCGGTCAGTA | Forward |
|  | CTGCTGGAGACTCAACATGC | Reverse |
| C9 | CTGGTCAGGATGGTGAACCT | Forward |
|  | TCAAATCCTCCTGACGCACT | Reverse |
| COMT | TGTGGAGTTTGTTGGCAGTG | Forward |
|  | TTACCTCCGTCAACACCGAA | Reverse |
| F2 | GGCAAAGAGGGCTAACACTG | Forward |
|  | TCTCGAGCTTCCTCATGGTC | Reverse |
| FGG | TGCCAACCTTACCAGAGGAG | Forward |
|  | AATCTCCGCAGTGTCTTTGC | Reverse |
| PLG | TGTGCAGGAATCCAGATGGT | Forward |
|  | CACCGCTCAATGTTGCAGTA | Reverse |
| SERPINF2 | AGCCAAGCAAAGTGTCACAG | Forward |
|  | TCCCATCCTCCATCTCCTGA | Reverse |
| TDO2 | ACAGGAAGCCACTCTCCTTC | Forward |
|  | CCCTCTTCGATGTTGGCTTG | Reverse |
